# Assessment of fluid biomarker profiles across disease continuum in a non‐human primate model of sporadic CAA

**DOI:** 10.1002/alz.092521

**Published:** 2025-01-09

**Authors:** Sohail Karimi, Akash G Patel, Pramod N Nehete, Bharti P Nehete, Ludovic Debure, Fengzhu Wang, Raul Rocha Botello, George Chahine, Qiao Zhang, Yongzhao Shao, Michele Marie Mulholland, Stanton B Gray, William D Hopkins, Thomas Wisniewski, Henrieta Scholtzova

**Affiliations:** ^1^ NYU Grossman School of Medicine, New York, NY USA; ^2^ UT MD Anderson Cancer Center, Michale E. Keeling Center for Comparative Medicine and Research, Bastrop, TX USA; ^3^ New York University Langone Health, New York, NY USA

## Abstract

**Background:**

Vascular Contributions to Cognitive Impairment and Dementia (VCID) is the second most common cause of dementia. Cerebral amyloid angiopathy (CAA), as one of the vascular pathologies underlying VCID, often coexists with Alzheimer’s disease (AD). The New World non‐human primate species, squirrel monkey (SQM), is a preclinical model of AD pathology that naturally develops extensive age‐associated CAA, and therefore holds immense translational value to study biomarkers and novel therapeutic approaches for AD and CAA. We aimed to assess the validity of fluid biomarkers and an Automated Cognitive Testing System (ACTS) to monitor disease progression in SQMs.

**Method:**

Plasma and CSF biomarkers of AD pathogenesis and neurodegeneration, cerebrovascular dysfunction/BBB integrity, and inflammation were characterized cross‐sectionally using SIMOA, ELISA, and Luminex immunoassays. A touchscreen‐based ACTS was implemented within young and geriatric SQM cohorts to assess behavioral performance.

**Result:**

Simple linear regression models revealed increases in plasma GFAP, sTrem2, YKL‐40 (CHI3L1), and both CSF and plasma NfL concentrations across the SQM lifespan. Plasma concentrations of MMP1 and MMP2 decreased with age, while no simple linear trends in MMP3 or MMP9 were observed. By applying segmented regression analyses, we estimated changepoints in plasma NfL, GFAP, neurogranin, MMP1, MMP2, MMP3, kallikrein‐6 (neurosin), as well as CSF NfL concentrations, ranging from 13.7 to 19.4 years of SQM age. Furthermore, a preliminary analysis among select cohorts of geriatric and young animals demonstrated an age‐associated decrease in the calculated Aβ42/Aβ40 ratio and albumin index, among time‐matched CSF and plasma samples. Training on an ACTS match‐to‐sample (MTS) task revealed differences in cognitive performance, as young SQMs required significantly fewer trials than geriatric animals to achieve a passing criterion. Cognitive assessments continue on a series of more advanced behavioral tasks. Integration of all measures with neuroimaging correlates are underway.

**Conclusion:**

The present characterizations of biomarker trajectories may help identify pathology onset and disease progression in the SQM model. Further portrayal of this species will advance our understanding of CAA pathogenesis, promoting further discoveries of diagnostic biomarkers and novel drug targets.

